# Uretero-fallopian fistula after hysteroscopy fallopian tube embolization: a case report

**DOI:** 10.1186/s12894-018-0385-9

**Published:** 2018-08-22

**Authors:** Yumeng Zhang, Tianjia Ma, Guangfeng Shao, Zhiying Xiao

**Affiliations:** Second Hospital of Shandong University, 247 Beiyuan Road, Jinan, 250033 People’s Republic of China

**Keywords:** Uretero-fallopian fistula, Fallopian tube embolization

## Abstract

**Background:**

Uretero-fallopian fistula (UFF) is a very rare surgery complication which usually happens after surgeries of fallopian tube or ureter. There has been no report of interventional operations of fallopian tube causing UFF.

**Case presentation:**

A 41-year-old female received fallopian tube embolization for birth control. After that she noticed “clear vaginal discharge”. She neglected that symptom for 7 years, until a sudden onset of abdominal pain brought her to the ER. Retrograde ureterogram confirmed UFF and revealed severe hydronephrosis of the left kidney. She received left nephrectomy afterwards and recovered well, with no urine leakage from her vagina.

**Conclusion:**

UFF could be caused by interventional operations of fallopian tube, and could lead to severe consequences. The application of fallopian tube embolization should be carefully controlled.

## Background

Uretero-fallopian fistula (UFF) is a very rare surgery complication which usually happens after surgeries of fallopian tube or ureter [1]. UFF could lead to severe complications like hydronephrosis and infection, and poses a great challenge for surgical repair. As far as we know, all reported UFF cases were caused by major surgeries like endometrioma removal, rectal cancer resection and open ureterolithotomy. There has been no report of interventional operations of fallopian tube causing UFF.

Fallopian tube embolization is a birth control method once widely used in China. It uses erosive media to block fallopian tube. This method has relatively high rate of complications like fallopian tube perforation, ectopic pregnancy and pelvic adhesion, and is currently being phased out. However, there has been no report of UFF caused by this method.

## Case presentation

**Fig. 1:**
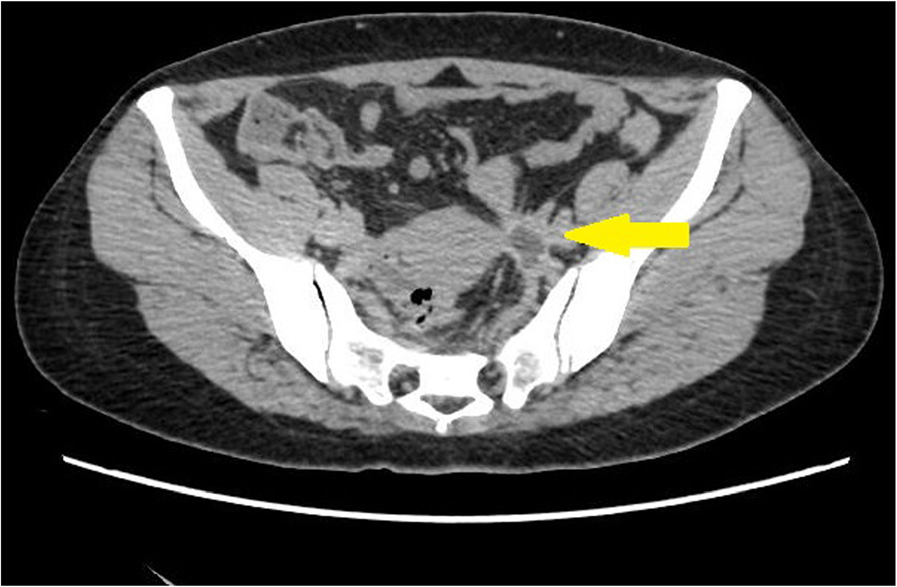
Pelvic CT scan showed a thick-walled cystic mass (arrow). The left ureter could not be clearly identified

On December 3rd, 2016, a 41-year old woman presented to the ER with colic pain in lower left abdomen. She had tenderness in her lower left abdomen, with no fever or hematuria. Emergency CT scan showed a thick-walled cystic mass (size: 2.1 × 1.5 cm) in the region of left adnexa (Fig. 1). The adjacent left ureter could not be clearly identified, and left proximal ureter dilated. It also revealed severe hydronephrosis on the left kidney with a very thin cortex. She was referred to urology department for further investigation.

Her past medical history was significant only for bilateral hysteroscopic fallopian tube embolization in 2009. It was an interventional birth control method. Four months later, she began noticing small amount of “clear vaginal discharge” which periodically started 3–5 days before period and ended in the last day of period. In the following 2 years, she underwent multiple gynecologic ultrasound exams and a hysteroscopy exam, but nothing abnormal was found. The patient didn’t seek further treatment, until the sudden occur of abdominal pain.

**Fig. 2:**
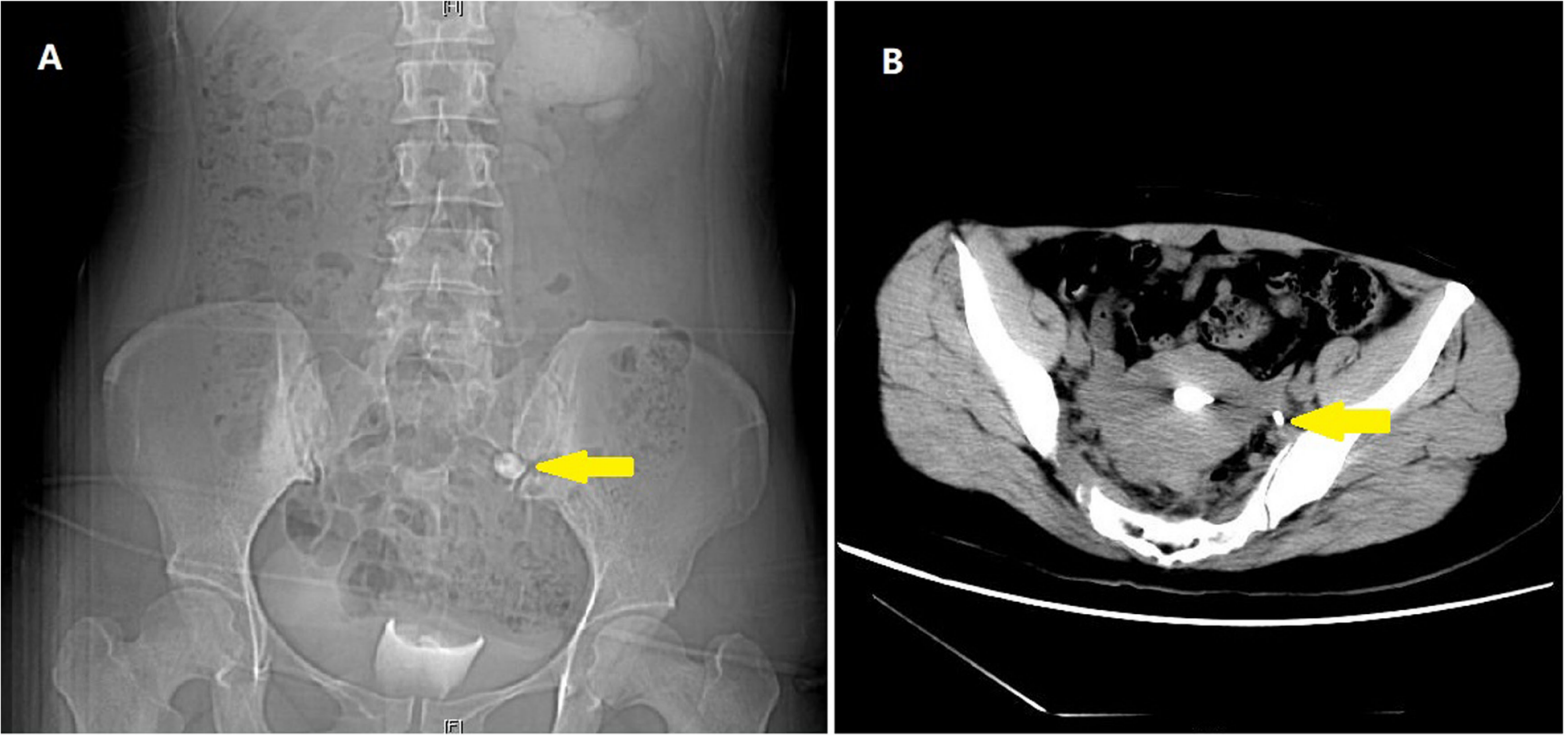
Retrograde CT ureterogram. **a** Contrast media reached left proximal ureter and left pelvis (Arrow: uretero-fallopian adhesion). **b** Contrast media was seen in uterus (Arrow: uretero-fallopian adhesion)

At our institution, she received various imaging exams. Gynecology ultrasound reported multiple myomas and otherwise nothing abnormal. To find the reason of hydronephrosis, we performed CT retrograde ureterogram. The exam showed that contrast media could reach left proximal ureter and pelvis (Fig. 2a), but extravasation of contrast media into the uterus could be clearly seen (Fig. 2b), confirming the presence of uretero-fallopian fistula. Consulting gynecologist performed hysteroscopy but no fistulous opening in the uterus could be seen. Given the fact that the glomerular filtration rate of her left kidney was less than 10 ml/min, left nephrectomy was carried out. We found her left ureter closely adhered to the fallopian tube and iliac artery during operation, so we only removed proximal ureter, leaving the distal part untouched. Her postoperative course was uneventful without vaginal discharge, and her creatinine level remained normal.

## Discussion

Ureteral injuries are most commonly iatrogenic in origin, typically during gynecological or urological surgeries [1]. Usually the urine leakage is localized or only diffuses into the peritoneal cavity, and uretero-fallopian fistula is very rare. Few cases reported UFFs were caused by major surgery procedures like endometrioma removal, rectal cancer resection and open ureterolithotomy [2–5]. Interestingly, in this case, UFF was caused by an interventional procedure-fallopian tube embolization, and led to severe hydronephrosis and nephrectomy.

Fallopian tube embolization was firstly designed as a birth control method in 1970s to meet the one-child policy in China. Comparing with traditional bilateral ligation, it was minimally invasive, quicker and easier, and quickly became popular in China. In 2016, Chinese couples were allowed to have two children, and therefore the application of this birth control method was significantly reduced. To our knowledge, in English literature there is no report of fallopian tube embolization except for an animal study using rabbits in 2001 [6].

Fallopian tube embolization could be done under hysteroscopy or fluoroscopy, or without imaging guidance at all. Commonly used embolic agent was a mixture of phenol, atabrine and iodipamide. Phenol serves as an erosion agent, atabrine stimulates the growth of granulation tissue, iodipamide is a contrast media that helps localizing the embolization site, and also helps stimulating granulation tissue, which effectively blocks the fallopian tube [7].

Previous studies reported that the success rate of fallopian tube embolization varied between 88.7 and 94.8%. It had similar success rate with bilateral tubal ligation, and had no risk of incision infection. Acute pain and fever may occur after fallopian tube embolization, chronic pain, irregular menstruation, uterus perforation and ectopic pregnancy have been reported during long-term follow-ups [8]. This method has one serious drawback: the standard dosage of embolic agent was never established, and it was possible for extra corrosive agents to perforate the fallopian tube. The extravasation of embolic agents might lead to adhesion between adjacent tissues and organs in pelvis. Laparoscopic examinations revealed that many patients had significant pelvic adhesion formation after fallopian tube embolization [9]. In our case, it was most likely that the embolic agent used caused fallopian tube perforation, and the extravasation of embolic agent caused the erosion of ureter and the adhesion of adjacent tissue, resulting in ureter stricture, uretero-fallopian tube fistula and left hydronephrosis.

UFFs are very rare complications which usually happened after major surgery procedures. In this case however, the UFF was secondary to an interventional procedure-fallopian tube embolization, and eventually led to the removal of a kidney. This rare and unusual case warned us that interventional operation of fallopian tube could also lead to severe complications. Increased awareness of fallopian perforation secondary to embolization should prompt hysteroscopy or fluoroscopy guidance, and careful evaluation of embolic agent dosage.

## Conclusions

Fallopian tube embolization using erosive media could cause severe complications, including very rare uretero-fallopian fistula. The application of fallopian tube embolization should be carefully controlled.

## Abbreviation

UFF: Uretero-fallopian fistula
